# Long-term management of leadless pacemakers

**DOI:** 10.1093/eurheartjsupp/suae119

**Published:** 2025-03-24

**Authors:** Andrew Andreae, Alexander Breitenstein, Jonathan P Piccini

**Affiliations:** Electrophysiology Section, Duke Heart Center, Duke University Medical Center, Box 31115, 2301 Erwin Rd, Durham, NC 27710, USA; Electrophysiology, Department of Cardiology, University Heart Center, University Hospital Zurich, Zurich, Switzerland; Electrophysiology Section, Duke Heart Center, Duke University Medical Center, Box 31115, 2301 Erwin Rd, Durham, NC 27710, USA

**Keywords:** State-of-the-art review, Leadless pacemaker, End of life, Retrieval, Explant, Extraction, Removal, Abandonment

## Abstract

Leadless pacemakers (LPs) are increasingly being used to treat bradyarrhythmias, and end of service (EOS) management decisions are becoming increasingly important given finite battery lifespans. Two strategies have been adopted for device EOS: LP abandonment and LP removal. Certain scenarios including high degree of LP encapsulation and ‘last expected’ pacing devices favour LP abandonment, while other LP complications may necessitate device removal. When abandoning LP, clinicians must understand design, performance, and safety considerations for devices left in place. When removing LP, specialized tools and techniques have been developed, each varying by device manufacturer and model. Case reports and series on LP removal have elucidated how to overcome challenges that may arise during removal. Despite best practice techniques, complications can arise before and after removal. Current studies on LP EOS management are limited, and further studies are needed to help understand predictors of successful device removal and long-term sequelae of both strategies. The aim of this state-of-the-art review is to help clinicians understand current strategies and considerations for both LP abandonment and removal.

## Introduction

Leadless pacing is increasingly being used to treat bradyarrhythmias due to low complication rates, growing indications, and evolution of leadless devices technology. There are now a large number of leadless pacemakers (LPs) in service across the world and end of service (EOS) management decisions will become increasingly important in the next 5 years. Given finite battery lifespans, many patients will require multiple LPs over their lifespan. End of service considerations are also important in situations where patients may require cardiac implanted electronic device (CIED) upgrade, as well as the occurrence of device malfunctions or infection.

Over the past 10 years, two strategies have been adopted for device EOS: LP abandonment and LP removal. The first LP to receive FDA approval, the Micra LP (Medtronic, Minneapolis, MN, USA), was designed to either be programmed off in a nonfunctional mode and abandoned at EOS or be removed. There are circumstances, such as device embolization and infection, which have necessitated removal of Micra LP utilizing the designed retrieval knob. While abandonment may be the preferred management strategy at EOS, depending upon the dwell time and the degree of fibrosis, removal may be reasonable at the time of EOS.^1,2^ The Nansotim/Aveir atrial and ventricular LPs (Abbott Medical, Sylmar, CA, USA) were designed to be removed at EOS utilizing a dedicated retrieval system.^3^ However, there is limited real world experience with AVEIR abandonment and removal in mid- and long-term follow-up given recent approval.

While the 2017 Heart Rhythm Society (HRS) Consensus statement (reaffirmed in 2022) is clear with respect to guidelines for explant of traditional CIED, current guidelines do not provide any unique recommendations or considerations for LP.^4^ Thus, the purpose of this review is to summarize the available data and provide guidance for clinicians who are confronted with managing LPs at EOS.

## Lifecycle of leadless pacemakers—endothelization, battery longevity, and performance at elective replacement indicator

To help select the optimal EOS strategy (LP abandonment vs. removal), it is helpful to understand the lifecycle of LPs, especially with respect to device encapsulation, battery lifespan, and elective replacement indicator (ERI) performance.

Case series on extracted Micra and Nanostim LPs and post-mortem pathologic examination of hearts with LPs shows that over the LP implant time, there is a fibrotic response to LPs leading to varying degrees from none to complete encapsulation (*Table 1*).^5–12^ This fibrosis matures from initial fibrocellular to later fibrosclerotic tissue.^5^ With the newer AVEIR LP System, encapsulation with fibrosis was shown to at least surround the distal fixation helix mechanism in ovine models, and in a human case report at least some portion of the atrial and ventricular LP length.^3,14^ Histopathologic examination of chronically implanted LPs reveals fibrosclerosis without chronic inflammation and thus encapsulation likely reaches a steady state during the device lifespan.^5^

**Table 1:** Case series and case reports describing leadless pacemaker encapsulation

Study	Publish year	Type of study	Patients (n)	Implant duration (months)	Device	Macroscopic tissue coverage	Maximal encapsulation thickness	Histology findings	Other key findings
Blessberger *et al*.^7^	2023	Autopsy, histology	8	mean 24 (range 0.5–70)	Micra	Partial (6/8), complete (2/8)	0.5–1.2 mm	Fibrous capsules and lymphocyte inflammation	LP encapsulation was not time-dependent
Kypta *et al*.^11^	2015	Autopsy, histology	1	12	Micra	Complete	—	Dense inflammation and collagenous capsule	Device ‘firmly adhered’ to RV and adjacent papillary muscle
Tjong *et al*.^12^	2015	Autopsy, histology	1	19	Nano-stim	Partial (60%)	<1 mm	Fibrous capsule and stabilized thrombus	—
Vamos *et al*.^6^	2016	Autopsy	1	3	Micra	Partial (66%)	not measured	—	—
Borgquist *et al*.^13^	2016	Autopsy	1	4	Micra	Complete	—	—	—
Satoh *et al*.^9^	2018	Autopsy	1	2	Micra	Partial	2 mm	—	—
Bonner *et al*.^8^	2022	Autopsy, histology	1	38	Micra	Near complete (95%)	1.1 mm	Peridevice fibrosis	Retrieval possible even with near complete encapsulation

In the vast majority of patients, device EOS will be at end of battery life (EOL). The estimated battery lifespan on LP varies by device model, spanning 13–18 years. The Micra VR1 and AV1 incorporate a 120 mAh battery, which in a real-world analysis of battery usage of 26 698 patients, the median projected battery longevity is 13.7 years (IQR: 12.4–15.3 years).^15^ The Medtronic AV2 and VR2 incorporate a 142 mAh battery, with manufacturer projected longevity of 15.6 and 16.7 years, respectively.^16^ The AVEIR ventricular LP incorporates a 243 mAh battery, with projected mean battery longevity of 17.6 ± 6.6 years when used in VVIR (95% CI: 16.6–18.6).^17^ The AVEIR atrial LP incorporates a 174 mAh battery, with projected battery lifespan median yet to be formally published in the literature but based on technical specification documentation is likely between 10 and 18 years when used in AAIR.^18^ Projected battery lifespans are lower when the AVEIR system is utilized in dual-chamber DDDR mode, likely between 6 and 12 years for each individual LP.^18^ Future generations of LP may incorporate higher energy density batteries to improve lifespan and prolong EOL.

Performance of LPs between their ERI and battery EOS varies between devices and manufacturers. With the Micra LP, once the device reaches the recommended replacement time, it enters into a defined 6-month prolonged service period (PSP). For the first 90 days of PSP, the device functions as previously programmed. After 90 days of PSP, the devices reaches ERI, and it automatically changes the pacing mode to VVI with pacing rate of 65 bpm and turns off rate responsiveness. After the 180-day PSP expires, the devices permanently switches to the device off mode.^19^ For Aveir ERI functionality, it is the same for both the ventricular and atrial LP. The devices automatically change to a pacing rate of 65 bpm and turn off rate responsiveness. The PSP varies between 6 and 12 months based on utilization of DDDR pacing, pacing amplitude, and impedance.^18^

## Conditions leading to end-of-service

Immediately following LP implant, patients are typically monitored post-procedure with telemetry, chest radiograph, and follow-up interrogation to detect acute device complications warranting early EOS. These early complications include elevated capture threshold or loss of capture after tether removal, partial or complete dislocation, tine fracture or helix damage, LP embolization, malfunction, and acute arrhythmias due to the device.^2,20–23^ If these are experienced, operators have typically retrieved the LPs, and either implanted a new LP or alternative pacing device.

In outpatient follow-up, clinicians monitor for late system complications via remote and in-person interrogations. Late system complications are infrequent but can include pacing failure, advisories, and rising or elevated thresholds that may warrant declaring a device at EOS (abandonment or removal).^2,20–23^ Beyond device failure, patients may develop pacemaker syndrome due to lack of AV synchrony, an indication for ICD or CRT upgrade, right ventricular pacing induced cardiomyopathy, arrhythmias, or in rarely right ventricular outflow tract (RVOT) obstruction. Any of these scenarios may lead to an EOS that could require abandonment or device removal.

**Figure 1:**
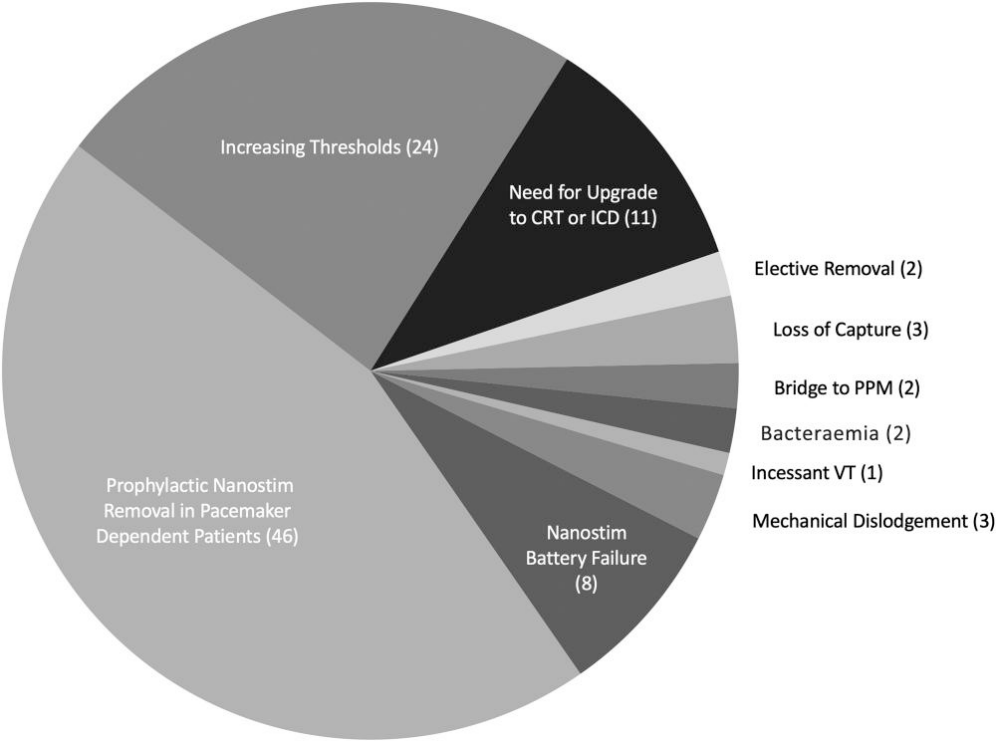
Prevalence of end-of-service aetiologies. The leading causes for leadless pacemaker removal in cross-sectional and registry data set historically was related to device advisory and battery failure of Nanostim leadless pacemaker. Excluding these, increasing device thresholds and need for upgrade to CRT or ICD has been the next most prevalent drivers of leadless pacemaker removal.

The relative frequencies of varies EOS aetiologies have been shown by combining data from Nanostim Leadless II post-trial registry along with direct manufacturer data for Micra TPS removal (*Figure 1*).^21,22^

## Terminology—explant, retrieval, extraction, removal, abandonment, and retainment

With transvenous devices, the HRS consensus statement has defined the term *explant* for device removal with a dwell time <1 year and *extraction* for removal when the dwell time is >1 year or those situations in which additional tools are required to remove the lead beyond those found in an implant kit.^4^ Prior LP literature has utilized these same time cut-offs.^24^ Consistent with predominant use of *retrieval* over *explant* in LP literature, we will utilize the term *retrieval* for explant <1 year and *extraction* for explant >1 year. Of note, case series and case reports show partial LP encapsulation at 2 months post-implant and complete encapsulation by 4 months (*Table 1*), and thus it is likely additional tools and equipment as discussed below will be required for extraction in as early as a few months following implant.

The term *removal* is used when discussing both LP retrieval and extraction. Both the terms *abandonment* and *retainment* have been utilized interchangeably for leaving LP in place at EOS, but we prefer the term *abandonment*, given its predominant usage in the literature.

## Choosing abandonment vs. removal

**Figure 2:**
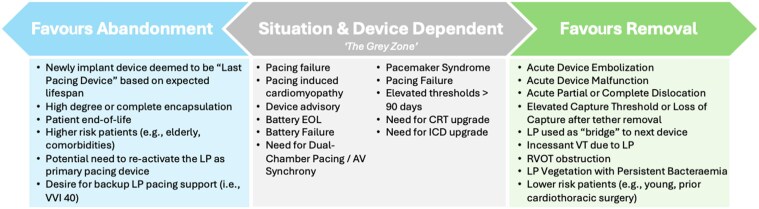
Factors favouring abandonment vs. removal. While certain clinical scenarios warrant leadless pacemaker removal (listed on right) and others favour leadless pacemaker abandonment (listed on left), some scenarios can utilize either strategy based on the clinical scenario.

When approaching a LP at EOS, clinicians must decide on a management strategy for the LP. In clinical studies at EOS, clinicians chose device removal in 36–52% of cases, while opting for device abandonment in 48–64% of cases.^20,22^ Certain scenarios favour removal or abandonment, while other scenarios are situational and device dependent (*Figure 2*).

### Scenarios favouring leadless pacemaker removal

If LP failure is discovered during implant procedure or immediately during the implant index hospitalization, retrieval is the preferred strategy since the device is free of encapsulation. Acute device-related complications within 30 days have been shown to occur in approximately 1.5% of LP implants.^25^ Causes can include elevated capture thresholds, fractured or damaged fixation mechanisms, loss of capture, and micro or macro-dislodgement.^26–30^ Device dislodgment or embolization has been shown to occur in approximately 0.29% of Micra VR implants and 0.88% of AVEIR VR implants and should also be managed with retrieval as device migration can be associated with several adverse events.^31^ Acute success with retrieval should be very high and a favourable strategy.

**Figure 3:**
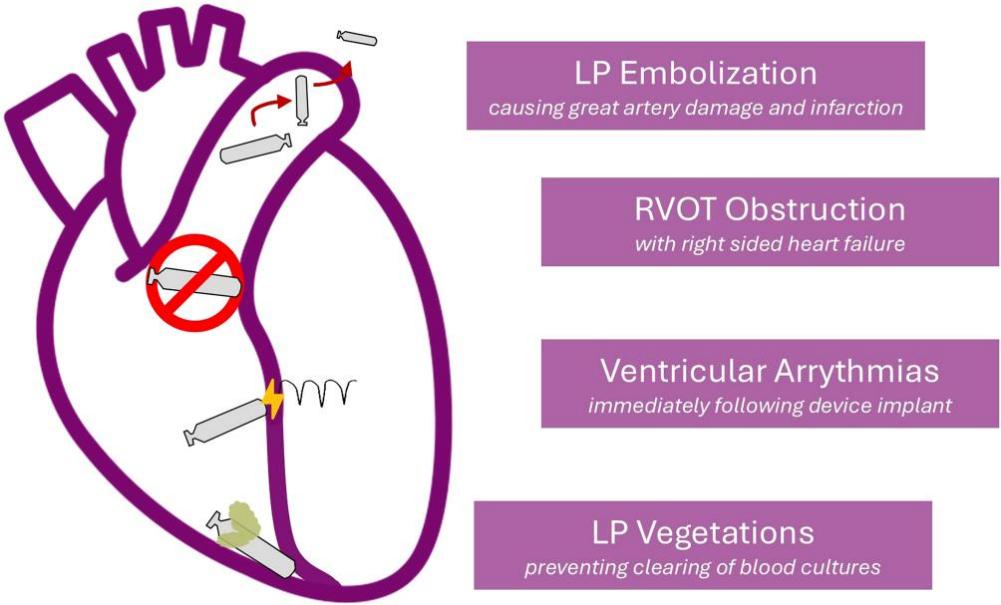
Life-threatening complications necessitating leadless pacemaker removal. Leadless pacemaker can cause certain life threatening complications, and thus warrant leadless pacemaker removal to alleviate the underlying pathology.

In the rare scenarios where the LP has caused a life-threatening complication (*Figure 3*), removal should remain the preferred strategy at EOS. In rare instances, LPs have been shown to cause sustained ventricular arrhythmias (VT and PMVT) with resolution after removal. Thus, urgent removal is the preferred treatment strategy for LP-induced arrhythmias.^26,27,32–35^ LP implant in the RVOT has rarely caused right-sided heart failure from RVOT obstruction, with removal immediately resolving obstruction and reducing heart failure symptoms.^36^ In situations of device embolization, the LP foreign body within pulmonary vasculature has put patients at significant risk of pulmonary infarction and great vessel damage, warranting removal.^37–39^ Patients with persistent bacteraemia despite antibiotics with vegetations seen attached to the LP should also undergo LP removal.

When a LP is used as a short-term bridge to next device (e.g. LP as bridge to new CRT-P following lead-associated endocarditis), removal is generally preferred to eliminate risk of infection of the new device and minimize intracardiac hardware.^20^ This is in contrast to when a LP is used as destination therapy following lead-associated endocarditis, where LP have appeared to be safe for reimplantation in patients with active CIED infection undergoing transvenous lead extraction.^40^ In young patients who are expected to need multiple pacemakers over their lifespan, LP removal may be favourable to minimize intracardiac hardware. However, the benefits and risks of extraction after long-dwell times may outweigh the risks of retained LPs with or without encapsulation.

### Scenarios favouring leadless pacemaker abandonment

In some scenarios, LP abandonment is the preferred strategy. When a LP is at EOS, and a newly implanted pacemaker is considered to be a ‘last pacing device’ with low likelihood of generator exchange or device upgrade, abandoning the old LP is likely a favourable strategy due to expected minimal complications with only one abandoned device.^20^

During attempted LP removal, if it is found that the LP is likely highly encapsulated or significantly adhered to the subvalvular tricuspid valve apparatus, the LP should be abandoned to reduce risks of major periprocedural complications including myocardial avulsion and valvular damage. Intraprocedural intracardiac echocardiography (ICE) may be particularly useful to help make this assessment. When a LP reaches EOS for a reason other than battery EOL, one strategy is to abandon the LP, leaving the ability to reactivate the LP as a primary pacing device or use as a backup pacer. In scenarios where a backup device is desirable, abandonment should be considered.

In patients deemed to be high risk for removal, abandonment should be favoured. There are a number of well-known risk factors for complications following the transvenous lead extraction including lead implant duration, age, low body weight, corticosteroid use, and female sex; however, it is unclear if these characteristics are also associated with complications following LP removal.^41^ While it is very likely that most risk factors for complications of extraction are common to both LP and transvenous devices, the relative importance of individual risk factors and the frequency of the complications may differ significantly. Future work should identify and validate risk factors for major complications of LP removal.

In many scenarios, both abandonment and removal may be reasonable strategies, and the decision should be dependent on patient risk factors for removal (e.g. age, comorbidities), degree of encapsulation, LP implant duration (retrieval vs. extraction). These scenarios are listed in *Figure 2*.

### Management of endocarditis with a leadless pacemaker

Leadless pacemaker infections are rare. There are a number of factors that reduce the risk of infection with LPs, including the absence of subcutaneous pocket, low surface area compared to transvenous systems, parylene-coating of the titanium device, encapsulation of the device over time, turbulent right ventricle (RV) fluid haemodynamics, and no physical handling of the LP at device implant.^42^

Overall infection rates have been evaluated in post-approval registries and other observational cohorts. In the Micra PAR, among 1809 patients implanted with Micra VR between July 2015 and March 2018, valvular endocarditis with possible LP involvement was seen in 1 patient (0.06%) over a 5-year follow-up.^2^ This infection resolved with IV antibiotics and without device removal. In fact, no devices were removed for infection in the post-approval registry. In a single-centre study of 302 Micra, 2 LP removals were performed for aortic and tricuspid valve endocarditis without LP involvement, and no Micra were extracted due to concern for infection of the Micra itself.^20^

While rare, infection of a LP can occur. There are a total of 7 case reports describing LP infections complicated by persistent bacteraemia and device vegetations despite intravenous antibiotics.^43–49^ In these reports, 6 of 7 underwent LP removal, with subsequent clearing of blood cultures. One out of 7 patients was transitioned to comfort measures without LP removal. In 4 of 6 cases, patients underwent debulking of the LP vegetations via percutaneous aspiration prior to LP removal. Given the potential consequences of incomplete treatment of a CIED infection, we recommend LP removal in situations of LP vegetations with persistent bacteraemia. Aspiration of large vegetations with one of several commercially available aspiration systems may be helpful to minimize the risk of embolization during the removal procedure.

When evaluating a patient with a LP and bacteraemia without a LP vegetation, the optimal approach is not defined. Some data suggest that LP may resist seeding with bacteraemia with or without valvular involvement. Leadless pacemakers have been implanted in at least 89 patients with active bacteraemia during transvenous lead extraction procedures, without subsequent LP infection during follow-up.^40^ Similarly, endocarditis has been managed conservatively without LP removal with subsequent clearing of cultures.^2^ The current 2017 HRS Consensus statement on CIED extraction recommends CIED removal in situations of positive blood cultures and valvular vegetations seen on transesophageal echo (TEE).^4^ Likewise, these guidelines recommend CIED extraction in settings of positive blood cultures, negative TEE, and specific microbiology including *Staphylococcus aureus*, coagulase negative *Staphylococcus*, and *Candida* species. Thus, current HRS consensus statement guidelines would recommend LP removal in situations of valvular endocarditis or bacteraemia with the above species. There is a need for larger studies to help clarify best practice for bacteraemia in persons with LPs.

## Leadless pacemaker abandonment

At end-of-life, leaving LP *in situ* is a common strategy. In the Micra-post approval registry (n = 1809), among those devices that reached EOS, 72 micra were left *in situ*, while 9 were percutaneously extracted.^2^ When a decision is made to abandon a LP, there are several important considerations for management.

### Programming of abandoned leadless pacemaker

The most common approach is to program the LP to device off mode (OOO). This ability to turn ‘off’ the LP is a unique feature not available in transvenous pacemakers. An alternative is to program the LP as a back-up pacemaker with a slower lower rate limit than a newly implanted pacemaker. For example, in the Micra PAR, 66 patients with devices left *in situ* were programmed to OOO, while 4 patients were programmed with back-up pacing (e.g. VVI 40).^2^

### Safety of abandoned leadless pacemaker

All currently available LP are magnetic resonance imaging (MRI) conditional when programmed on.^18,19^ Similarly, an abandoned LP programmed to OOO is not a contraindication to MRI. With traditional transvenous CIED, MRI is considered non-conditional with abandoned leads due to induction across the lead and radiofrequency-induced lead tip heating. Given leadless nature of LP, this is not of concern with abandoned LP (similar to the situation with implantable loop monitors).

Abandoned LP likely have inconsequential effects of cardiac haemodynamics. The volume of current LP is small (0.8–1.1 mL), and when compared to average right ventricular end diastolic volume in men of 163 and 127 mL in women by cardiac MRI, occupy < 1% of RV volume, and thus have limited effects on haemodynamics.^50–52^ Long-term risks >10 years of LP dwell time are yet to be determined as the first LP were implanted in 2014. Observational studies and registries are needed to monitor for potential unanticipated long-term risks of LP abandonment.

### New pacemaker implant with abandoned leadless pacemaker

Three concerns have been raised related to implanting a new LP while abandoning an existing LP: (i) device–device mechanical interference, (ii) device–device electrical interference, and (iii) mechanical effects in the RV related to multiple LP devices.

Device–device mechanical interactions have not been observed in post-approval registries but has been reported in at least one case report.^53^ In the Micra PAR 5-year follow-up, amongst 13 patients who had a second Micra device within the RV while abandoning the first Micra, there were no adverse events related to device–device interactions.^2^ Amongst 1423 Nanostim LP implanted worldwide, following the St Jude (now Abbott Medical) advisory, there were 115 patients where LPs were abandoned and a new device was implanted next to the abandoned LP, with no adverse device-to device interactions reported. Similarly in a single-centre experience of 302 Micra implants, 12 patients had their LP abandoned at EOS, with no interactions between the new pacing system and abandoned LP during follow-up.^20^ However, mechanical interference can occur during a LP implant procedure.^53^ In this case, a Micra LP was implanted immediately adjacent to an abandoned Micra (programmed OOO), with subsequent constant noise and ventricular oversensing events believed to be due to physical contact between the two devices. This interference was managed with prompt retrieval of the older device. Similar to implanting a new RV lead in the presence of an abandoned transvenous lead, operators should utilize a different implant location to avoid LP device-device contact during the cardiac cycle. For example, if the abandoned LP was implanted in the septal RV apex, operators should implant the new device in the proximal or mid RV septum.

Electrical interference is a theoretical concern, but this risk should be eliminated when the abandoned LP is programmed to OOO mode. To our knowledge, no cases of electrical interference have been documented regarding LP abandoned in a backup pacing mode (i.e. VVI 40) with a new device. Thus, theoretical electrical interference is generally of low concern.

Regarding mechanical effects of multiple LP, preclinical studies suggest that the human RV can accommodate up to 3 LPs without physical interaction or touching, even with small patients with relatively small RV chamber (35 mL).^54^

## Leadless pacemaker removal

### Planning for leadless pacemaker removal

In preparation for LP removal, operators should review the LP implant records, consider pre-procedural imaging (chest x-ray, CT, MRI, or TEE), develop a primary removal strategy, be prepared for ‘bail out’ techniques, and arrange cardiothoracic surgery backup when appropriate. Operators should also then decide on an ‘implant then explant’ or ‘explant then implant’ strategy.

Periprocedural imaging can be particularly helpful to define the location and orientation of the device and advanced imaging modalities like CT, MRI, or CT may help elucidate possible encapsulation and relationship and/or binding to the tricuspid apparatus and moderator band. For patients receiving a new CIED device at the time of removal, two procedural options are available—‘implant then explant’ or ‘explant then implant’ strategy. The advantage of implanting prior to explant is to have new pacing in place prior to device removal; however, this is limited by the potential to dislodge the new device in situations of difficult removal. Utilizing an explant first strategy prevents operators from accidentally dislodging a new device, however, may require additional femoral access for temporary intracardiac pacing.

In cases of LP retrieval (<1-year dwell time), it should be performed in an electrophysiology lab in a hospital where cardiothoracic surgery is available. In cases of LP extraction (>1 year), extraction should be performed with immediate cardiothoracic surgery backup in a hybrid operating room. Cardiothoracic surgery backup in a hybrid operating room should also be arranged with a dwell time <1 year when cases are deemed to be higher risk. Examples of such situations include free wall placement, history of a pericardial effusion during the original implant, or encapsulation seen on imaging.

### Removal techniques

Snaring the LP via a femoral vein approach is the main strategy used for removal of a LP. All commercially available LPs have a proximal retrieval button designed to be snared by a single- or triple-loop snare. Moderate sedation or general anaesthesia has been utilized based on anticipated procedure length and expected difficulty.

In all cases, ultrasound guided access is typically obtained via the right or left femoral vein. Ultimately, the vein is then dilated to 27 mm utilizing either the dedicated retrieval system or dedicated implant introducer sheath. This sheath is used for ultimate export of the LP device out of the body; however, additional sheaths may be inserted into the export sheath. For example, a deflectable 8-Fr sheath can be very helpful directing snares within the RV. Superior approaches via the right internal jugular have been utilized for LP removal and can be useful when an inferior approach is unsuccessful for snaring the LP docking feature or when the patient’s size or anatomy is unsuitable for an inferior approach.^55,56^ Additional venous access for temporary pacing should also be obtained in pacemaker-dependent patients or those with a left bundle branch block where trauma to the right bundle could result in complete heart block.

Biplane fluoroscopy and intraprocedural echocardiographic imaging can also be helpful during LP removal. Intracardiac echocardiography or TEE can be utilized to visualize LP orientation, LP movement, and monitor for complications (e.g. pericardial effusion and tricuspid valve damage). In addition, ICE has been utilized to identify encapsulation and additional tissue around the LP at time of removal that may make the procedure less successful. Further, ICE may help visualize whether the docking button is accessible via snare and freely moving within the RV.

**Figure 4:**
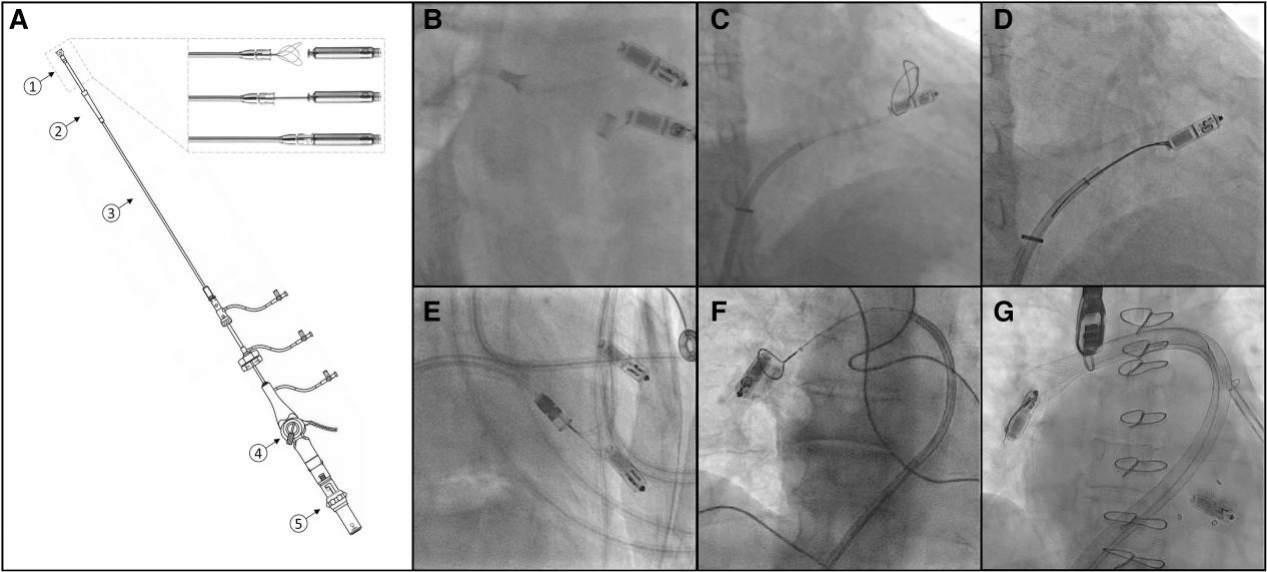
Removal devices and techniques. (*A*) Demonstrates the AVEIR Retrieval System with docking system and tri-loop snare, with (1) tri-loop snare, (2) protective sleeve, (3) guide catheter, (4) deflection lever and brake, and (5) snare control knob and handle. (*B*) Demonstrates snaring a Micra through a Micra delivery system. (*C* and *D*) Demonstrates retrieving a Micra utilizing a steerable sheath (Agilis) and snare. (*E*) Shows retrieval of a Micra utilizing the AVEIR Retrieval System. (*F*) Demonstrates Micra retrieval from the pulmonary artery utilizing a multipurpose catheter and a 10 mm GooseNeck snare. (*G*) Demonstrates capture of leadless pacemaker from the pulmonary artery utilizing the ONO basket retrieval system. Procedural images (*B*–*F*) are courtesy of author Dr. Breitenstein Alexander, and image *G* is courtesy of Dr Aron Bender of University of California, Los Angeles.

Choice of sheath and removal setup is dependent on the manufacturer and model of LP due to variable external diameters of the LP cylindrical shape and thus is detailed separately below and in *Figure 4*. When utilizing a non-designated sheath for removal, it should be confirmed prior to the case that the sheath has adequate internal diameter to retrieve the LP body.

#### Removal of Abbott Nanostim and AVEIR devices

Abbott Medical has manufactured dedicated removal systems for its two LP devices—the Nanostim LP, which was discontinued in 2016 due to premature battery depletion, and the newer AVEIR single and dual chamber LP, which is their redesign and replacement for the Nanostim. The Nanostim retrieval catheter system (Abbott Medical) with integrated snare was developed to remove the Nanostim LP by unscrewing the fixation helix from the myocardium and recapturing the device. This system is no longer in production and has been replaced by the newer AVEIR retrieval system (Abbott Medical), which is recommended for explanting older Abbott Nanostim LP.^57^

The AVEIR retrieval catheter system with integrated tri-loop snare is a single operator steerable system designed for removal of AVEIR atrial and ventricular LP (*Figure 4A*). After venous access is obtained, the system is advanced to the right atrium (RA) and then across the tricuspid valve towards to LP utilizing the integrated deflector for steering. After snaring, the LP retrieval feature, the LP is re-docked, and the sleeve can be advanced over the LP to provide countertraction against the surrounding myocardium with the goal of reducing myocardial avulsion.^58^ During advancement of the protective sleeve, if there is a resistance, outpouching, or billowing of the protective sleeve, it likely suggests tissue entrapment (e.g. tricuspid valve apparatus) or LP encapsulation and the operator should avoid further advancement. Due to the helix design of the fixation mechanism, the snare is rotated counterclockwise while observing 3 full turns of the LP radiopaque marker on fluoroscopy. The AVEIR retrieval catheter system is compatible with the docking features of the Micra, Modular LP (Boston Scientific), and the sleeve has an internal diameter able to accommodate these LPs.^58,59^

#### Removal of Boston Scientific modular leadless pacemaker

The Modular LP (Boston Scientific, Marlborough, MA, USA), which is currently still under investigation as of December 2024, also has a dedicated retrieval catheter with integrated snare. It also utilizes a snare to snag the retrieval feature, re-dock the LP, and allow advancement of a recapture sheath over the LP.^60^

#### Removal of Medtronic Micra devices

The Micra VR, VR2, AV, and AV2 do not have a dedicated retrieval system but were developed to utilize an off-the-shelf snare combined with the 27 Fr Micra introducer sheath. Three approaches have been utilized for removal of Micra LP.^24,61^

In the first approach (*Figure 4B*), the Micra introducer system is advanced into the RA and then RV in the typical fashion for Micra implantation under fluoroscopic guidance.^62^ The delivery cup is positioned as close to the proximal aspect of the Micra as possible. Then, a 5–7 mm single-loop goose-neck snare is advanced through the delivery catheter and around the proximal retrieval button. The snare is cinched to lock to LP. Then, the combination of traction on the snare and countertraction from the cup will release the tines from the myocardium, and the entire system with snared LP can be fully retracted into the introducer sheath. This technique may be favourable when implanting a replacement Micra in the same procedure. In this scenario, the same delivery sheath used to deploy the LP is used to snare the old Micra.^24,61,63^ Another advantage is the ability to provide counter-traction from the device cup. However, this technique is restricted by the limited steerability of the Micra delivery catheter (unidirectional) and maximal snare size (7 mm) that can be advanced through the delivery catheter.^61^

In the second approach (*Figure 4C*, *4D*), femoral vein access is obtained utilizing the 27-Fr Micra introducer sheath. A 14–16 F sheath is then placed inside the 27 F sheath to prevent back bleeding from the haemostatic valve (sheath in sheath technique). Then, a steerable sheath (e.g. Abbot Agilis NxT or Medtronic Cryoablation Sheath) is inserted and used to advance across the tricuspid valve and as close as possible to the Micra. A 20–30 mm large loop or multiloop snare (e.g. Amplatz Goose Neck 20 mm snare, Medtronic or EnSnare, Merit Medical) is inserted through the steerable sheath, and utilizing multiple fluoroscopic view, encircled around the LP body. The snare is then withdrawn slowly and cinched around the retrieval feature. The steerable sheath is then advanced while retracting the snare to lock the device, and the complete assembly is brought back into the 27 F introducer sheath.^24,61^ In some cases, operators experience resistance while removing the steerable sheath and LP through the haemostatic valve of the 27 F sheath, which has required the whole assembly including the 27 F sheath to be removed en-block as a system.^21^ Generally, this second approach allows for additional steerability and larger snares, which makes snaring LPs easier. This technique is restricted by the inability to provide countertraction as the steerable sheath cannot be advanced over the LP.

In the third approach, the AVEIR retrieval catheter system has been utilized to remove Micra pacemakers utilizing the same procedure detailed above for AVEIR LPs (*Figure 4E*).^58,59^ This technique allows for both steerability and countertraction.

#### Removing free-floating and embolized leadless pacemaker

Embolized LP may become completely free-floating in the RA, RV, or coronary sinus.^26,32,38,64–66^ Devices have also embolized anterogradely to the pulmonary arteries or retrogradely into the inferior vena cava or femoral veins. In situations where the device is free floating, 2 snares are typically utilized either through a 1 or 2 sheath system. In this ‘double snare technique’, one snare is typically utilized to capture the LP body or tines allowing for stabilization of the actively mobile device. The second snare is utilized to capture the proximal retrieval feature to allow the device to be brought co-axially into the introducer sheath. Snaring the device anywhere on the LP should be done as quickly as possible as to avoid LP embolization to the pulmonary artery or damage to intracardiac structures.^38^ Once the device is captured, it can always be snared with additional tools in the inferior vena cava to help achieve a coaxial alignment. In situations where the retrieval feature cannot be snared due to the LP orientation, the LP tines can been snared and the LP removed via an ‘upside-down’ technique.^55^ Further, in situations where two snares from an inferior approach cannot adequately mobilise the LP, a two-directional approach with the second snare placed via the internal jugular vein may be more succesful.^32^

Leadless pacemakers have embolized to the pulmonary artery in at least 9 case reports.^37,39,61,67–72^ In these situations, a steerable sheath as described above will not be long enough to reach the LP. Multiple techniques have been described for removal from the pulmonary vasculature. In the first technique (utilized in 7 of 9 cases), a multipurpose catheter should be advanced over a guidewire in the pulmonary artery (*Figure 4F*). Then, the guidewire can be removed, and a snare can be advanced through the multipurpose catheter and onto the body of the LP. Multiple snares may be required to manoeuvre the LP within the pulmonary vasculature. Depending on LP device orientation following embolization (retrieval vs. fixation feature first), snaring the tines may be required in the pulmonary artery, with device re-orientation in the right heart or inferior vena cava to facilitate recapture via the retrieval knob.^39^ When the LP cannot be snared, a second technique utilizing the ONO endovascular retrieval system (ONOCOR LLC, Philadelphia, PA, USA) with nitinol expandable basket-like trap has been used to recapture the LP (*Figure 4G*).^69^ In a third technique, the INARI Flowtriever system (Inari Medical, Irvine, CA, USA), which is a 24 F sheath typically used for suction thrombectomy of pulmonary emboli, can be used. In this method, the Flowtriever catheter is directed towards into the pulmonary artery, and further traditional gooseneck snares are utilized to stabilize the LP and recapture it into the Flowtriever catheter.^37^

In situations of ‘bailing out’ embolized LPs, a team-based approach with assistance from cardiothoracic surgery, interventional radiology, interventional cardiology, and/or cardiac anaesthesia may be pivotal to achieving successful outcomes.^37^

#### Removal during open heart surgery

In the setting of infective endocarditis or other planned open-heart surgery, LPs have been removed surgically under direct visualization.^2,73^ In order to avoid myocardial avulsion associated with excessive traction force on the LP, a counter-traction technique utilizing a 2.5 cc syringe and silk thread has been utilized.^73^ This countertraction on the surrounding myocardium is like the countertraction provided by the AVEIR retrieval catheter system. Surgical removal can be considered in patients necessitating explant after failing percutaneous explant or undergoing cardiac surgery for other reasons.

### Overcoming challenges during leadless pacemaker removal

#### Ensuring coaxial alignment

When capturing a LP during device removal, it is impossible to withdraw the LP into the retrieval sheath if it is oriented perpendicular to the retrieval catheter, as the LP must be coaxial. In a situation where the LP is off-axis, 2 snares can be used to grasp different portions of the LP (e.g. the body and the retrieval feature), in order to successfully orient the LP and guide it into the retrieval sheath.^74^ Confirming coaxial removal is best done by reviewing the orientation of the snare with the retrieval features utilizing orthogonal x-ray views.^56,61^

#### Inability to engage the proximal retrieval feature

In situations where the snare can be placed around the LP body, but upon retraction and tensioning is unable to grab the retrieval feature, this might suggest that the retrieval feature has become encapsulated.^75^ As snaring the retrieval feature is essential for removal success, this situation typically requires abandonment of the LP.

It has been proposed one could utilize a Bioptome or Raptor (Diagmed Healthcare, North Yorkshire, UK) forceps catheter to cut and expose the proximal portion of the LP.^74^ However, to our knowledge, there are no case reports of this technique being utilized *in vivo*. Such manoeuvers would be considered very high risk and would necessitate CT surgical back-up.

#### Tines fixed on intracardiac structures

Case reports of LP removal have experienced the exposed tines of Micra LP hooking onto structures including the tricuspid valve apparatus and myocardium possibly causing injury.^55^ In this situation, the tines were released from the tricuspid valve by binding them together with a snare. This technique of tightening a snare around the four exposed tines has also been utilized to retract tines inadvertently puncturing through central vasculature walls.^37^

#### Encapsulation and adhesions

The rates and temporal kinetics of LP adhesion formation and the subsequent degree of encapsulation have not been formally investigated; however, their presence has been demonstrated in case series and reports. Irretrievable LPs have no swinging motion on fluoroscopy, suggesting either dense adhesions or significant encapsulation.^76^ Likewise, 2 Nanostim LP removals were complicated by new significant tricuspid regurgitation, likely from adhesions to the tricuspid valve apparatus.^22^ In another case series of 15 Nanostim removals, 2 of 3 unsuccessful removals were attributed to docking button location within the tricuspid valve apparatus with adhesions.^75^ In a histopathologic examination of 15 Nanostim LP removals, tissue was present on 14 of 15 explants, with one patient tissue consisting of tricuspid valve tissue and chorda tendinea, with follow-up echocardiograph demonstrating significant increase in TR.^5^ Encapsulation and adhesions encountered during the LP removal attempt may require LP abandonment.

If an operator experiences difficulty snaring the retrieval feature, utilizing ICE or contrast fluoroscopy may help clarify if encapsulation tissue, adhesions, or the subvalvular apparatus is blocking access.^76^

### Safety of leadless pacemaker removal

Successful device removal has been defined as complete removal of the LP during the removal procedure. The removal success rate for LPs ranges between 87 and 100% in clinical studies, and a variety of complications have been observed (*Table 2*).^2,20–22,27,77^ Complication rates range from 0 to 5.5%.^2,20–22,27,77^ No risk scores have yet to be created with regards to LP removal risks and success. Other risks of LP removal include those typical for cardiac catheterization including vascular access complications, bleeding, haematoma, air embolism, local and systemic infection, anaesthesia complications, amongst many others. Given large femoral sheaths used, access site bleeding can be controlled by utilizing vascular access closure devices or applying prolonged pressure.

**Table 2:** Studies investigating leadless pacemaker removal**^a^**

Study	Publish year	Type of study	Device	Removal attempts (*n*)	Removal success, *(n*, %)	Implant duration	Indications for removal	Complications (*n*, %, reasons)	Reasons for Unsuccessful Removals
Lakkireddy *et al*.^22^^a^	2017	Post-Approval Registry	Nanostim	73	66 (90.4%)	Range 0.2–4.0 years	Battery failure (8), prophylactic removal due to advisory (46), device upgrade (9), elective removal (2), high thresholds (8)	4 (5.5%), 1 docking button detachment, 1 arteriovenous fistula, 2 tricuspid valve damage causing TR	4 docking button inaccessible, 1 docking button detachment
Afzal *et al*.^21^	2018	Manufacturer Registry	Micra TPS	29	29 (100%)	Range 1–61 days	Immediate high threshold/loss of capture (8), immediate dislodgement (3), delayed high threshold (11), infection (2), need for transvenous device (2), incessant VT (1)	0 (0%)	n/a
Bhatia *et al*.^20^	2021	Single-centre experience	Micra TPS	11	11 (100%)	Median 78 days (IQR 14–113)	High threshold (3), pacing induced cardiomyopathy (3), bridging to next device (3), endocarditis without LP involvement (3)	0 (0%)	n/a
Reddy *et al*.^77,a^	2021	Multicentre Clinical Trial Registry	Nanostim	201	176 (87.6%)	Mean 2.6 ± 1.5 years (range 0 to 7.2)	Not specified	10 (5.0%), 3 tricuspid valve injury, 2 inability to release snare from docking button, 2 docking button detachment, 3 not specified	19 docking button inaccessible, 2 docking button detachment, 2 inability to deliver the retrieval catheter, 1 inability to remove the snare, 1 inability to unscrew the LP
Garg *et al*.^27^	2023	FDA MAUDE Database Query	Aveir LP	15	15 (100%)	Not specified	Device dislodgment (10), high threshold (3), sustained ventricular arrythmia (1), fragmented tether remains (1)	Not specified	Not specified
El-Chami *et al*.^2^	2024	Post-approval registry	Micra VR	10	10 (100%)	Range 0.1–47.4 months	Device upgrade (3), high threshold (7)	1 (10%), 1 LP entanglement in inferior vena cava filter	n/a

^a^Studies are not mutually exclusive and may contain similar patient cohorts.

### Predictors of success for leadless pacemaker removal

Some studies have assessed predictors for LP removal success; however, the data are very limited. In a single-centre report of 34 Nanostim LP removals, a ‘swinging movement’ of the docking button of the LP > 15° under fluoroscopy was associated with successful removal. In the swinging motion group, removal rates were significantly higher than those without swinging motion (25 of 25 patients vs. 4 of 9 patients; *P* < 0.001^76^). Leadless pacemakers without swinging motion were more likely to be placed in the RV septum rather than the RV apex (*P=* 0.0016). It is possible that LPs implanted in the septum become more entrapped or adherent to the tricuspid subvalvular apparatus. In the 5 patients where removal was unsuccessful, the docking button could not be snared, and thus it was theorized that lack of swinging motion was probably due to adherent tissue/encapsulation possibly preventing access. In a separate review examining swinging motion, the LP with the smallest angle of swinging motion observed (3°) had tricuspid valve apparatus present on histopathologic examination of the LP and subsequent tricuspid valve damage.^5^ These small case series suggest LPs located in the septal apex and with docking buttons with significant swinging movement may have higher success with removal.

Given encapsulation that can occur around LP, it has been theorized that duration of implant may reduce success rates of removals. However, this has not been demonstrated in post-approval registries. Amongst 73 Nanostim removals, removal success rates were 86% at <1 year of implant duration, 93% at 1–2 years, and 90% at >2 years (*P* > 0.05). Hence, duration of implant was not associated with short term removal success.^22^ In a study on 15 attempted Nanostim LP extractions with longer implant durations (median device age of 1040 days), a lower extraction success rate of 80% was observed, and attributed to possible progressive encapsulation over time.^75^ More data are needed to evaluate the association of medium and long implant durations (e.g. >5 years) with explant success.

### Sequelae of leadless pacemaker removal

Following device removal, a tissue ‘cast’ or ‘sock’ has been visualized in cases via ICE and fluoroscopy with contrast.^5,78^ It has been hypothesized that this ‘cast’ represents encapsulation tissue that was covering the device left behind. It is unknown the prevalence of this phenomenon, and if there are any sequelae of this residual void.

Implanting a new LP immediately after removal appears to be safe without any reports of an increased risk of adverse events. In a case series of 35 Nanostim removals, 27 patients received no LPs during the same procedure immediately following LP removal, with no procedure-related complications during reimplantation procedures.

## Patient end of life—pacing continuation and cremation

Nearing end of life, patients may request deactivation of defibrillator tachytherapies (e.g. shocks) to improve patient comfort for hospice or withdrawal of support. However, deactivation of pacing therapies, including those from LP, is typically seen as active ending of life rather than withdrawal of support, and thus is not performed at patient end of life.^79^ Where LP may be used for antitachycardia pacing (ATP) in the future, one could deactivate ATP in line with goals of hospice and patient comfort. However, deactivating LP bradycardia pacing is not recommended.

Following patient death, the current standard of practice for cremating patients with transvenous pacemakers and defibrillators is surgical explant before cremation to prevent device explosion. In contrast, LPs are approved for cremation without the need for explant based on manufacturer bench, cadaveric, and real-world testing.^80,81^

## Implications for future practice

Recommendations for LP management when confronting the decision to abandon or remove a LP are summarized in *Table 3*. Recommendations for future research in *Table 4*.

**Table 3:** Recommendations for leadless pacemaker removal vs. abandonment

	Recommendation
1	Available case reports and series show no major safety risks for choosing a LP abandonment strategy at EOS, although data are limited to implants <10 years at time of this review.
2	Clinicians should favour a LP abandonment strategy when the newly implanted device is considered to be a ‘last pacing device’ based on life expectancy.
3	Clinicians should favour a LP removal strategy in situations of device embolization, device malfunction < 30 days, incessant arrhythmias due to the LP, elevated capture thresholds or loss of capture after tether removal, partial or complete dislocation < 30 days, young patients with need for multiple pacemakers over their lifespan, and LP use as a ‘bridge’ to next device.
4	In situation of ‘bailing out’ embolized or entrapped LPs, a team-based approach with assistance from cardiothoracic surgery, interventional radiology, interventional cardiology, and/or cardiac anaesthesia may be pivotal to achieving successful removal.
5	When implanting a new CIED while abandoning an existing LP, operators should utilize a differing implant location than that used for the LP to minimize device–device contact during the cardiac cycle.

**Table 4:** Important questions for future research

What are the lifetime effects and safety of single and multiple abandoned LPs?
Can imaging technologies be utilized to detect LP encapsulation, endothelization, and adhesions prior to LP removal? This would help facilitate pre-procedure planning, determining the need for cardiothoracic surgery backup, and expected procedure success.
What patient characteristics and device locations present the highest risk for LP removal?
What are the predictors of successful and unsuccessful LP device removal?
How can LP removal techniques and devices be simplified and improved?
How does LP implant location affect removal procedures?
What are the rates of LP removal success with medium to long implant durations (e.g. > 5 years)?
What are the risk factors for major complications of LP removal? Are they the same as the risk factors for transvenous lead extraction?

## Conclusions

End of service management of LPs is becoming increasingly important as the utilization of LPs increases and more devices begin to approach EOL. Initial experiences with LP demonstrate that abandonment and removal are viable strategies; however, certain scenarios warrant choice of one over the other. Whether or not LP will become the dominant pacing strategy in the future is up for debate, LPs will likely remain a fixture in the pacing landscape. Thus, management of LPs and the potential multiple LP implants over a patient’s lifespan will become increasingly important for clinicians. Further research and data are needed to help clinicians better understand risks of LP removal and abandonment.

## Data Availability

No new data were generated in support of this research.
